# p16 in highly malignant esophageal carcinomas: the correlation with clinicopathological factors and human papillomavirus infection

**DOI:** 10.1007/s00428-020-02865-x

**Published:** 2020-06-16

**Authors:** Hirotaka Ishida, Atsuko Kasajima, Fumiyoshi Fujishima, Ryujiro Akaishi, Shunsuke Ueki, Yuto Yamazaki, Yoshiaki Onodera, Xin Gao, Hiroshi Okamoto, Yusuke Taniyama, Takashi Kamei, Hironobu Sasano

**Affiliations:** 1 Department of Surgery, Tohoku University Graduate School of Medicine, Sendai, Japan; 2 Department of Pathology, Tohoku University Graduate School of Medicine, Sendai, Japan; 3 School of Medicine, Griffith University, Gold Coast, Australia; 4 Department of Pathology, Technical University Munich, Trogerstr. 18, 81675 Munich, Germany; 5 German Cancer Research Center (DKFZ), Heidelberg, Germany

**Keywords:** Esophagus, Small-cell carcinoma, Poorly differentiated squamous cell carcinoma, Basaloid squamous cell carcinoma, p16, Human papillomavirus

## Abstract

p16 is generally considered to be a surrogate maker of human papillomavirus (HPV) infection and also a predictive marker of favorable clinical outcome of patients with squamous cell carcinoma of the oropharynx. p16 overexpression is also known to be induced by deregulation of *RB1* in neuroendocrine carcinomas. In highly malignant esophageal neoplasms, however, the status of p16 has remained largely unknown. We immunolocalized p16 and Rb1 in 82 surgically resected esophageal high-grade squamous cell carcinomas (46 poorly differentiated and 36 basaloid squamous cell carcinomas) and 15 esophageal small-cell carcinomas in order to clarify the clinical and biological significance of p16. p16 immunoreactivity was detected in 7/82 (9%) high-grade squamous cell carcinomas and 15 (100%) small-cell carcinomas. p16 immunoreactivity was significantly associated with Rb1 protein loss in both groups (*P* < 0.001). HPV was detected in none of the p16-positive cases examined. Clinical outcome of the p16-positive high-grade squamous cell carcinomas was not different from that of the p16-negative counterparts (*P* = 0.687) but significantly better than those with the small-cell carcinomas (*P* = 0.023). p16 was therefore considered to be induced through an inactivation of the RB1 signaling pathway and not through HPV infection in highly malignant esophageal neoplasms. Nevertheless, patients’ clinical outcome of these neoplasms significantly differs; therefore, small-cell carcinomas have to be carefully differentiated from other neoplasms. In addition, p16 overexpression is not predictive of favorable clinical outcome in high-grade squamous cell carcinomas of the esophagus.

**Electronic supplementary material:**

The online version of this article (10.1007/s00428-020-02865-x) contains supplementary material, which is available to authorized users.

## Introduction

Approximately 85% of esophageal neoplasms are squamous cell carcinomas in East Asian countries [1, 2]. Among these patients, histological differentiation and types are associated with clinical outcome of the patients; in particular, poorly differentiated or basaloid squamous cell carcinomas of the esophagus have significantly worse prognosis than well- to moderately differentiated ones [3–5]. In addition, esophageal small-cell carcinomas, which account for 0.05–3.1% of all esophageal malignancies [1, 6, 7], are known to be highly aggressive [3, 6, 8].

p16 is a protein product of tumor suppressor gene *CDKN2A* and a member of the INK4 family of cyclin-dependent kinase inhibitors. p16 binds to and inactivates cyclin-dependent kinases 4/6, resulting in the suppression of the phosphorylation of retinoblastoma 1 (Rb1) protein (a tumor suppressor protein) and cell cycle progression [9, 10]. In general, *CDKN2A* is frequently inactivated in human malignant tumors by its gene aberrations, such as deletions or mutations [9, 10], but diffuse p16 expression was also reported in various human malignancies, e.g., high-grade breast and lung cancers and/or undifferentiated pleomorphic sarcoma without significant association with its gene alteration status [9, 11]. In these neoplasms, p16 was reported to be induced by deregulation of *RB1* as positive feedback [9, 12]. The p16 overexpression via *RB1* alteration was on the one hand demonstrated in highly aggressive tumors, such as small-cell carcinomas of the lung and esophagus [12–14]. In particular, p16 overexpression and loss of Rb1 protein were constantly detected in esophageal small-cell carcinomas [12].

On the other hand, p16 protein overexpression is also considered a surrogate marker of oncogenic human papillomavirus (HPV) infection. HPV infection plays a pivotal role in carcinogenesis of oropharyngeal, uterine cervical, and perianal cancers [9, 15, 16]. Following the entry of virion via endocytosis, the virus establishes a persistent infection as a viral episome or integrates into the host genome [17]. HPV E6 and E7 oncoproteins are expressed from both forms of the viral DNA, which could inactivate tumor suppressors p53 and Rb1 proteins and lead to cell cycle progression and subsequently to development of cancer [17, 18]. Consequently, an inactivation of Rb1 protein results in the overexpression of p16 [9, 12]. HPV-associated oropharyngeal squamous cell carcinomas were pathologically and clinically different from the non-HPV-associated counterparts [19]. These tumors had a lower metastatic rate and TNM stages than HPV-negative squamous cell carcinomas [19], resulting in better clinical outcome and response to chemotherapy, radiotherapy, or chemoradiotherapy than those with HPV-negative squamous cell carcinomas [20–23]. Therefore, an evaluation of p16 status was proposed essential in the diagnosis of oropharyngeal malignancy [24]. At this juncture, p16-positive oropharyngeal squamous cell carcinomas are separately staged from those without p16 expression [24]. In the esophagus, HPV-positive squamous cell carcinomas were also reported, but its reported incidence enormously varied: 0–63% in Japan, 1–77% in China, 8–74% in India, 0–41% in Europe, and 0–5% in the USA [25–28]. In addition, the patients with p16-/HPV-positive squamous cell carcinoma of the esophagus were reported to be associated with better clinical outcome [28, 29] and respond better to chemoradiotherapy than the p16-/HPV-negative patients [30]. However, the status of p16 and its correlation with HPV infection in highly malignant neoplasms of the esophagus have remained largely unknown.

In our present study, we aimed to clarify (i) the status of p16 and its relation with HPV infection, (ii) the clinicopathological characteristics of p16-positive tumors in comparison with p16-negative tumors, and (iii) the clinical outcome of the corresponding patients in highly malignant neoplasms of the esophagus including poorly differentiated and basaloid squamous cell carcinomas, as well as esophageal small-cell carcinomas.

## Materials and methods

### Tumor specimens

We studied 97 esophageal carcinoma cases: 15 small-cell carcinomas and 82 high-grade squamous cell carcinomas. The high-grade squamous cell carcinomas, termed based on their aggressive clinical nature, consisted of 46 poorly differentiated and 36 basaloid squamous cell carcinomas [3, 5]. All the specimens were surgically resected between 1988 and 2017. The specimens of esophageal small-cell carcinoma were retrieved from six Japanese institutions listed in a previous study [6], and high-grade squamous cell carcinomas from a single institution (Tohoku University Hospital, Sendai, Japan). The clinical data of the patients were reported in the previous studies to compare the outcomes of small-cell carcinoma and basaloid squamous cell carcinoma patients, as well as SOX2 and Rb1 expression in small-cell carcinomas [3, 6]. All the specimens were independently reviewed by three pathologists (AK, FF, and HS). The histological diagnosis of small-cell carcinoma was made based on the histomorphology and presence of immunoreaction of at least one of synaptophysin and chromogranin A [6, 31]. Pathological diagnosis of high-grade squamous cell carcinoma was made based on histomorphology and presence of positive staining for p40 by immunohistochemistry as previously described [3, 6, 31]. Tumors with the following conditions were excluded: (i) located at the esophagogastric junction or concomitant Barrett’s esophagus and (ii) eradication of more than two thirds of the tumor with preoperative therapy [6].

Clinical information of all the patients examined was carefully reviewed. TNM staging was determined according to the eighth edition of the American Joint Committee on Cancer/Union for International Cancer Control TNM staging system for esophageal carcinoma [24]. Disease-specific survival was defined as the time from initial pathological diagnosis to disease-related death or last observation. The study protocol was approved by the ethics committees of all participating institutions.

### Immunohistochemical analysis

Specimen preparation and immunohistochemical stainings of synaptophysin, chromogranin A, p40, and Rb1 were performed as previously described [6]. Anti-p16 monoclonal antibody (clone G175-1239; dilution 1:100, pH 6.0 buffer) was commercially obtained from BD Pharmingen, NJ, USA. The above-mentioned immunohistochemistry was performed in all cases included in this study. Labeling index against the nuclear antigens p40, p16, and Rb1 was all quantified using the HistoQuest semi-automated image analysis software (TissueGnostics, Tarzana, Los Angeles, USA) in an area of 0.04 mm^2^, and the percentage of immunopositive nuclei in total tumor cells was subsequently obtained [6, 32]. In evaluating synaptophysin and chromogranin A immunoreactivity, a well-established immunoreactivity scoring system ranging 0–12 was employed. The score was calculated by multiplying the positivity (0, 0%; 1, < 10%; 2, 10–50%; 3, 51–80%; or 4, > 80% of tumor cells positively stained) and the staining intensity (0, none; 1, mild; 2, moderate; or 3, strong staining), and positive immunoreactivity was defined as a final score of ≥ 1 and negative as a final score of 0, as previously described [6, 33]. Positive immunoreactivity of p40 and p16 was defined as their labeling index of ≥ 1% and negative as index < 1% [6, 33]. Positive (normal) immunoreactivity of Rb1 was defined as its labeling index of ≥ 10% and negative (loss) as index < 10% [34]. Reactive stromal cells, lymphocytes, and/or non-neoplastic epithelial cells were used as positive internal controls for p16 and Rb1 (Supplementary Fig. 1). The evaluation methods are summarized in Supplementary Table 1.

### DNA extraction from 10% formalin-fixed, paraffin-embedded blocks

In order to explore the status of HPV infection in p16-positive tumors, genomic DNA of both esophageal small-cell carcinoma and high-grade squamous cell carcinoma cases was selectively extracted from the tumorous area of unstained tissue sections (thickness, 10 μm × 5 sections) under the guidance of serial hematoxylin and eosin-stained tissue sections using a QIAamp DNA FFPE Tissue Kit (Qiagen, Valencia, CA, USA) according to the manufacturer’s protocol [35]. The quality of the extracted DNA was confirmed by polymerase chain reaction (PCR) using a beta-globin (human) Primer Set (TaKaRa Bio., Shiga, Japan) according to the manufacturer’s instructions [36]. An amplified DNA sample via PCR was considered as an appropriate sample for the following PCR experiment.

### Polymerase chain reaction for typing human papillomavirus

PCR was performed in samples using a Light Cycler equipment (Roche, Basel, Switzerland) and commercially available HPV typing set (TaKaRa Bio., Shiga, Japan) to identify HPV genotypes 6, 11, 16, 18, 31, 33, 35, 52, and 58 in genomic DNA with specifically designed primer sets and according to the manufacturer’s instructions [37]. Other rare genotypes of HPV were not detectable by this approach. The primer sequences used were as follows: HPV pU-31B forward for low-risk HPV genotypes 6 and 11, 5′-TGCTAATTCGGTGCTACCTG-3′; HPV pU-1M forward for high-risk HPV genotypes 16, 18, 31, 33, 35, 52, and 58, 5′-TGTCAAAAACCGTTGTGTCC-3′; and HPV pU-2R reverse, 5′-GAGCTGTCGCTTAATTGCTC-3′. This primer set included both malignant and benign control templates for verification of the PCR results using the primer pairs provided in the set. The PCR products were evaluated by 2.0% agarose gel electrophoresis stained with ethidium bromide in Tris–borate–ethylenediaminetetraacetic acid buffer.

### Statistical analyses

JMP Pro version 13.0.0 software (SAS Institute, Inc., Cary, NC, USA) was used for all statistical analyses. Continuous data were analyzed using Student’s *t* test or the Mann–Whitney *U* test. Correlations between two variables were identified using Pearson’s chi-square test, Fisher’s exact test, or Mann–Whitney *U* test as appropriate. Disease-specific survival curves were constructed using the Kaplan–Meier method, and compared using the log-rank test. A *P* value of < 0.05 was considered statistically significant.

## Results

### Clinicopathological features

The clinicopathological characteristics of the 97 esophageal neoplasms examined in this study are summarized in Supplementary Table 2. There were no significant differences in age, gender, tumor location, and tumor size between small-cell and high-grade squamous cell carcinomas or between poorly differentiated and basaloid squamous cell carcinomas (data not shown). The TNM stage of small-cell carcinoma was higher than that of high-grade squamous cell carcinoma (*P* = 0.018, data not shown).

### Immunohistochemical characteristics

**Fig. 1:**
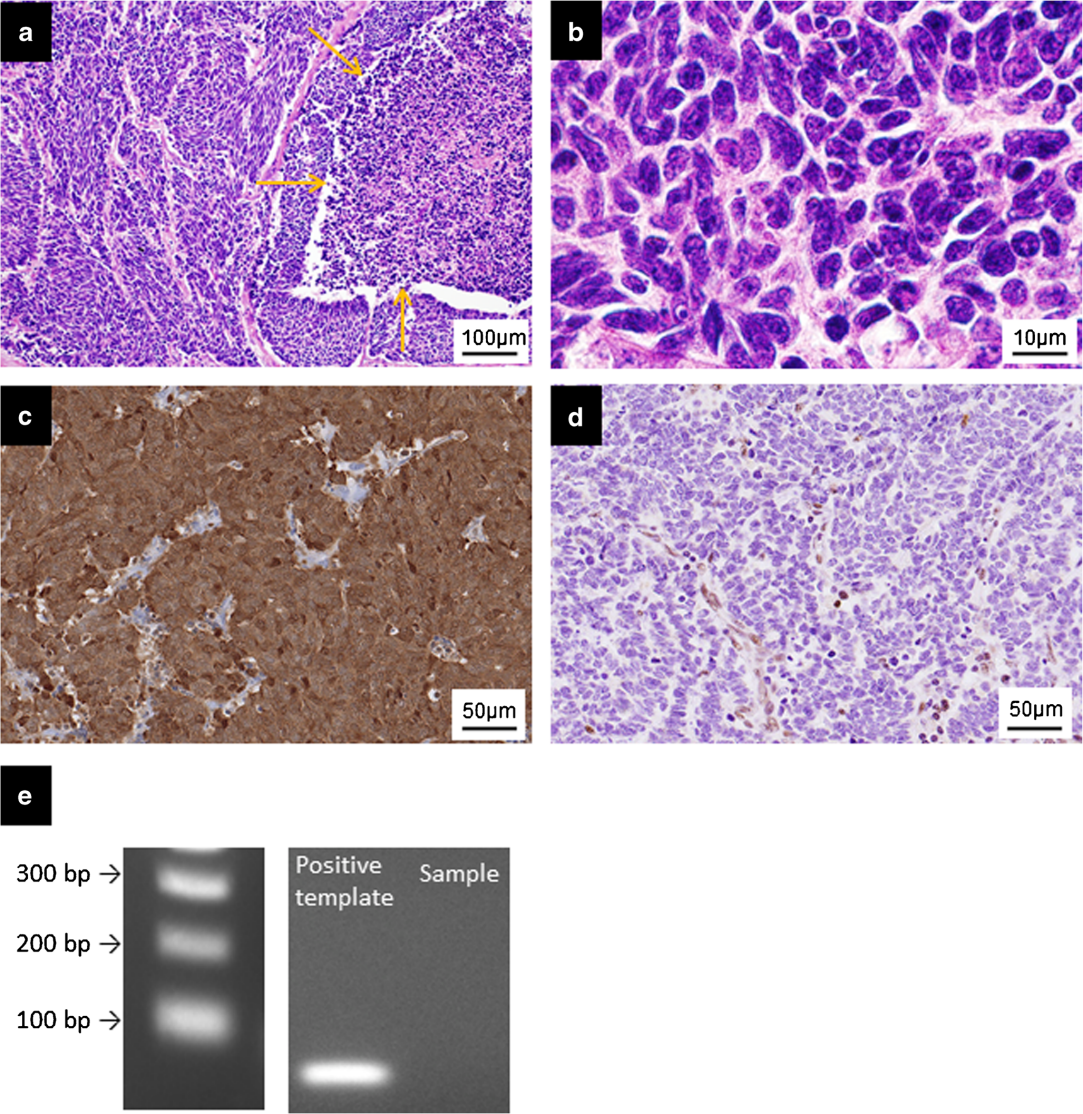
Histopathological and immunohistochemical features of esophageal small-cell carcinoma. The tumor with variably sized solid nests with ill border. Abundant intratumoral necrosis (yellow arrows, **a**). Tumor cells are round to oval in shape with hyperchromatic nuclei and scant cytoplasm (**b**). Diffuse nuclear and cytoplasmic p16 immunoreactivity (**c**) and loss of Rb1 protein in the carcinoma cells (**d**). No amplification of human papillomavirus DNA of a small-cell carcinoma tissue and a control tissue showing a positive band by polymerase chain reaction (**e**)

**Fig. 2:**
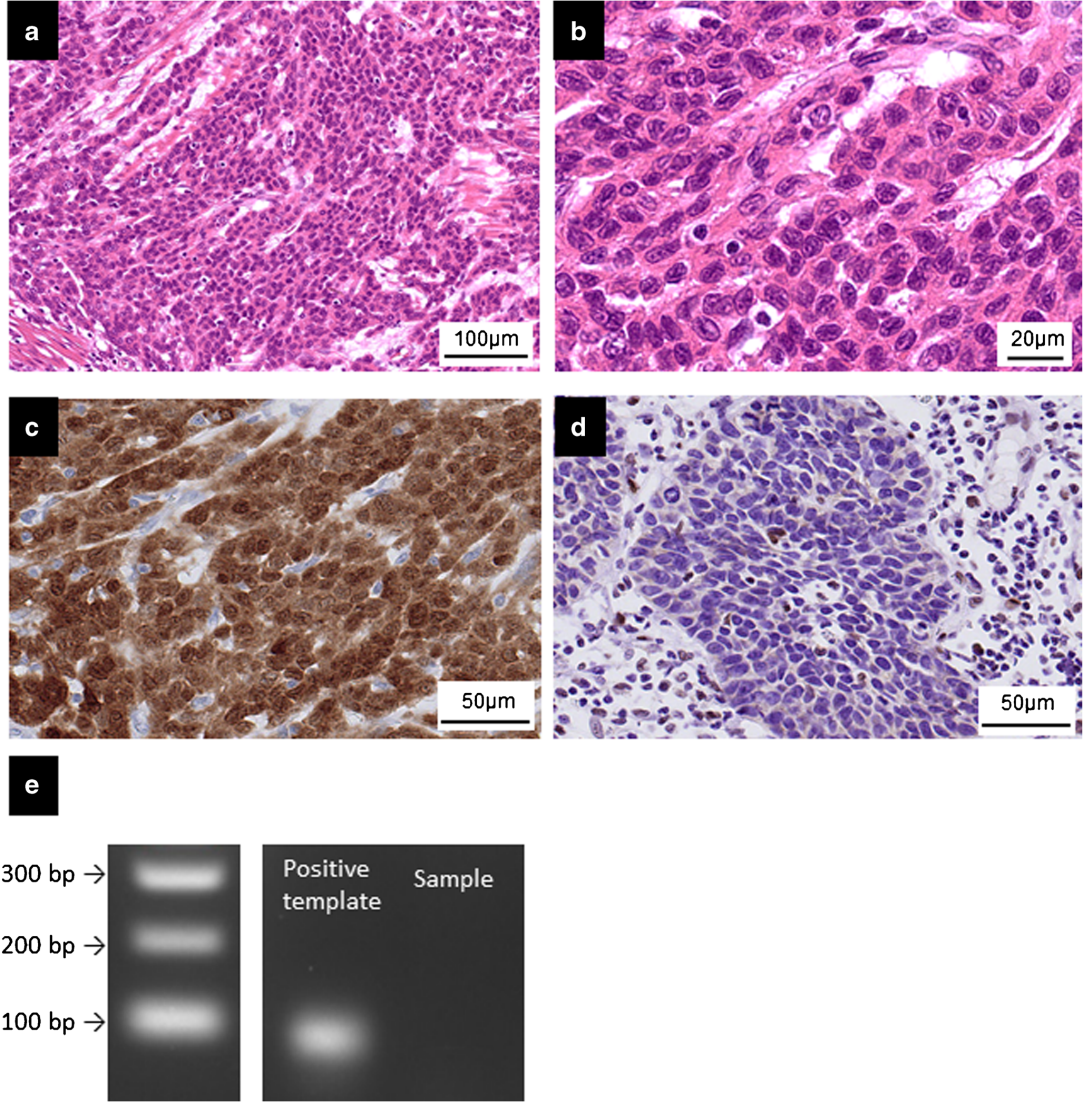
Histopathological and immunohistochemical features of poorly differentiated squamous cell carcinoma of the esophagus. Tumor cells of poorly differentiated squamous cell carcinoma comprised round cells without apparent keratinization (**a** and **b**). Immunoreactivity of p16 was expressed in 7 out of 82 cases (**c**), while loss of Rb1 protein was detected in 12 out of 82 cases of high-grade squamous cell carcinoma (**d**). No amplification of human papillomavirus DNA (**e**)

**Fig. 3:**
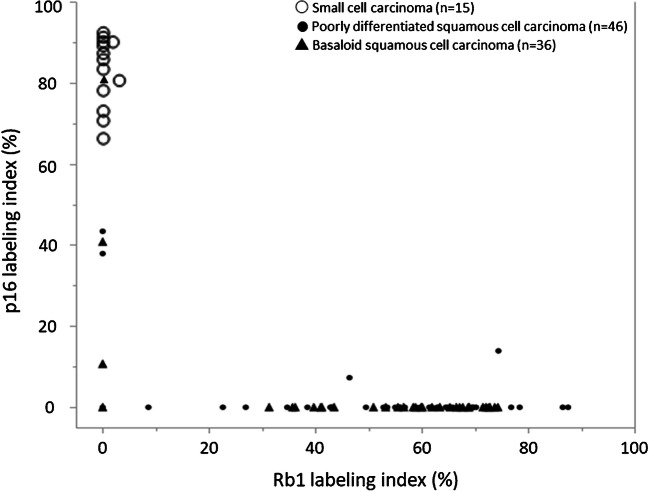
All the small-cell carcinomas (plotted in white circles) demonstrated high immunoreactivity of p16 and loss of Rb1 protein expression. In contrast, most of the poorly differentiated (black points) and basaloid (black triangles) squamous cell carcinomas showed no p16 overexpression and normal Rb1 expression. p16 expression and loss of Rb1 expression were observed in 4 and 3 cases of poorly differentiated and basaloid squamous cell carcinomas, respectively. The p16 expression was significantly associated with the loss of Rb1 protein (*P* < 0.001)

The results of the immunohistochemistry analysis of esophageal small-cell and high-grade squamous cell carcinomas are summarized in Table 1. Synaptophysin immunoreactivity was detected in all esophageal small-cell carcinomas, whereas chromogranin A was expressed in 10 (67%) esophageal small-cell carcinomas. Expression of synaptophysin and chromogranin A was not detected in any of the high-grade squamous cell carcinoma specimens. p16 expression was detected in all the small-cell carcinomas (*n* = 15, 100%). The immunoreactivity was observed mostly in nuclei and cytoplasm of the tumor cells with strong intensity and consistent high labeling index (range, 63–93%; mean, 83%; Fig. 1). In the high-grade squamous cell carcinomas, p16 immunoreactivity was focally detected in nuclei and cytoplasm with strong intensity in 7/82 cases (9%) (Fig. 2). The mean labeling index of the cases with positive p16 immunoreaction (7 cases) was 33%, ranging from 7% to 80% (Fig. 3). The p16 labeling index was also significantly higher in the esophageal small-cell carcinomas than in the poorly differentiated squamous cell carcinomas, as well as the basaloid squamous cell carcinomas (*P* < 0.001 for both) (Table 1). No significant difference was detected in p16 status between the poorly differentiated squamous cell (mean index 2% in all cases, 4/46 positive) and the basaloid squamous cell carcinomas (mean index 3% in all cases, 3/36 positive). All esophageal small-cell carcinomas exhibited diffuse and uniformly strong immunopositivity of p16, whereas high-grade squamous cell carcinomas demonstrated a diffuse and strong pattern in one case (1%), a focal positivity in 5 cases (6%), and a single-cell positivity in one case (1%). Loss of Rb1 protein expression was detected in all the 15 small-cell carcinomas (Figs. 1 and 3) and 12/82 of the high-grade squamous cell carcinomas (15%) (Figs. 2 and 3). In the high-grade squamous cell carcinomas, the status of p16 immunoreactivity was significantly associated with female gender (*P* = 0.047) but not with age, location, size, TNM stage, or histological type (Supplementary Table 3). The labeling indexes of p16 and Rb1 were inversely correlated with each other (*P* < 0.001, Fig. 3). In order to test whether the used cut-off level of 1% for determination of p16 expression status is appropriate to correlate Rb1 loss, we also tested different values (from 5% to 35%) but they did not result in different statistical correlations. Among the p16-positive high-grade squamous cell carcinomas (*n* = 7), 4 with a relatively high labeling index of p16 (index, 80, 43, 41, and 38%) demonstrated no Rb1 protein expression (index, all 0%) and presented features related to aggressive biological behavior: tumor size ≥ 50 mm, lymph node involvement, and a high TNM stage (III/IV). In contrast, the other 3 with a low labeling index of p16 (index, 7, 11, and 14%) demonstrated indolent clinical characteristics: tumor size < 50 mm, no lymph node metastasis, and a low TNM stage (I/II). Two out of the 3 had normal Rb1 protein expression (index, 74 and 46%) (Fig. 3).

**Table 1:** Immunohistochemical profiles of 15 esophageal small-cell carcinomas and 46 poorly differentiated and 36 basaloid squamous cell carcinomas

	Small-cell carcinoma *N* (%)	High-grade squamous cell carcinoma		*P* value
		Poorly differentiatedsquamous cell carcinoma *N* (%)	Basaloid squamous cell carcinoma *N* (%)	Small-cell carcinoma vs. high-grade squamous cell carcinoma
Total (%)	15 (100)	46 (100)	36 (100)	
Synaptophysin				
Pos	15 (100)	0 (0)	0 (0)	< 0.001
Neg	0 (0)	46 (100)	36 (100)	
Chromogranin A				
Pos	10 (67)	0 (0)	0 (0)	< 0.001
Neg	5 (33)	46 (100)	36 (100)	
p40				
Pos	1 (7)	46 (100)	36 (100)	< 0.001
Neg	14 (93)	0 (0)	0 (0)	
Mean ± SD (%)	4 ± 16	81 ± 15	73 ± 16	< 0.001
p16				
Pos	15 (100)	4 (9)	3 (8)	< 0.001
Neg	0 (0)	42 (91)	33 (92)	
Mean ± SD (%)	83 ± 10	2 ± 9	3 ± 14	< 0.001
Rb1				
Pos	0 (0)	40 (87)	30 (83)	< 0.001
Neg	15 (100)	6 (13)	6 (17)	
Mean ± SD (%)	0.3 ± 1	51 ± 24	48 ± 25	< 0.001

*Pos* positive, *Neg* negative, *SD* standard deviation, *Rb1* retinoblastoma 1

Marked and diffuse immunoreactivity of p40 was detected in all the high-grade squamous cell carcinomas (100%, 82/82 cases) with a mean index of 78%, whereas p40 was negative in 14 small-cell carcinomas. The other one small-cell carcinoma (7%, 1/15 case) presented weak p40 immunoreactivity.

We confirmed the accuracy of staining using controls’ tissues listed in Supplementary Table 1.

### Human papillomavirus status in p16-expressing neoplasms

DNA were extracted from 3 of the small-cell carcinomas with the highest labeling index of p16 (93, 92, and 91%) and all the high-grade squamous cell carcinomas with positive p16 expression. The extracted DNA was amplified using beta-globin (human) Primer Set. DNA amplification was not successful in one of the high-grade squamous cell carcinomas probably due to insufficient purity of the extracted DNA. We conducted PCR using the amplified DNA for detection of HPV infection. In both malignant control (63 bp) and benign control (61 bp) templates, the DNA was amplified and PCR was performed successfully. No amplification of HPV DNA regardless of tissue subtypes was detected in esophageal small-cell and high-grade squamous cell carcinomas (Figs. 1e and 2e).

### Clinical outcome

**Fig. 4:**
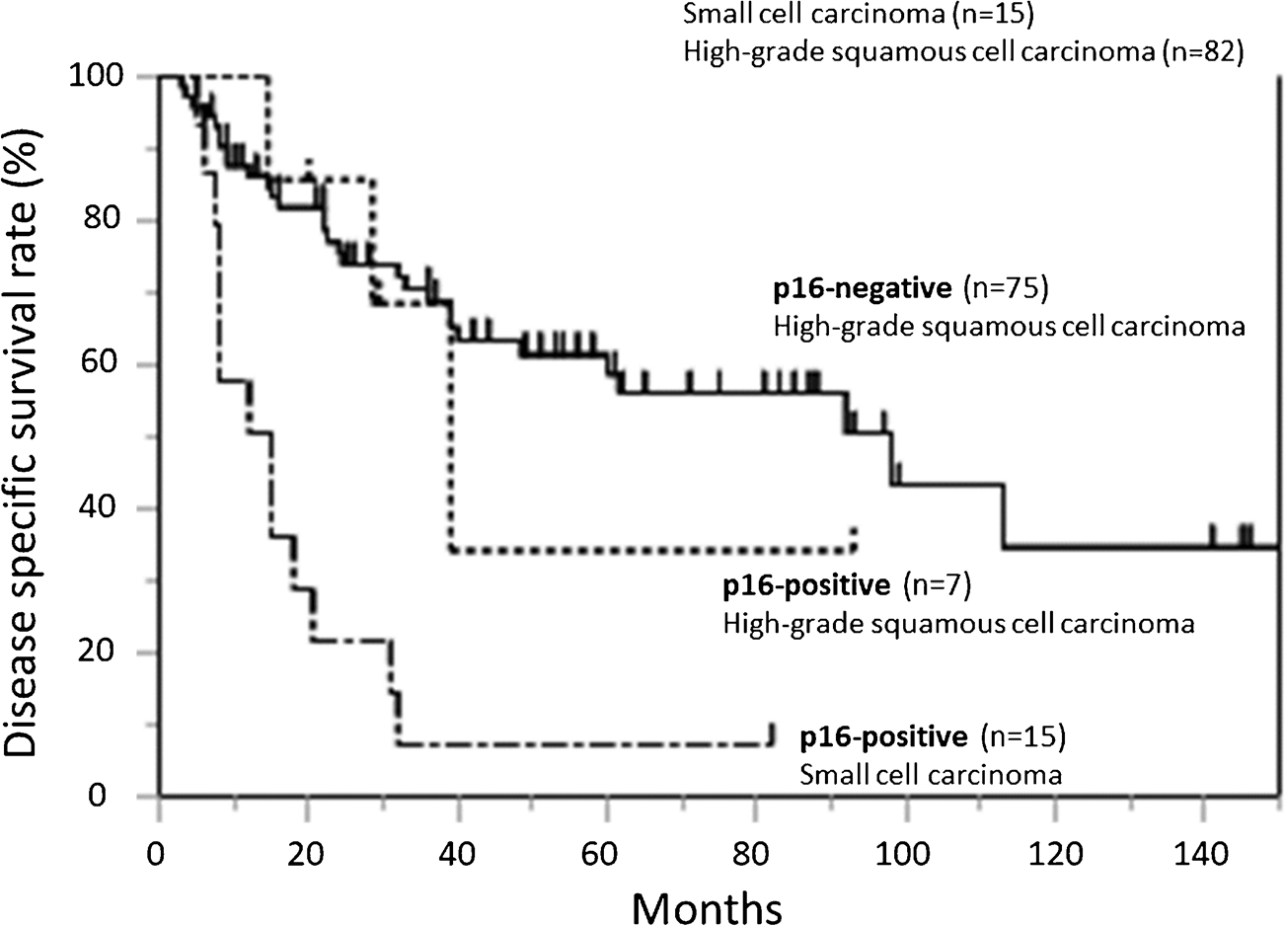
Clinical outcome of the patients. Disease-specific survival of the esophageal small-cell carcinoma patients was significantly worse than that of high-grade squamous cell carcinoma patients (*P* < 0.001). No significant difference was observed between the p16-positive and p16-negative high-grade squamous cell carcinoma patients (*P* = 0.687). Outcome of the small-cell carcinoma patients was also significantly shorter than that of the p16-positive and p16-negative groups of high-grade squamous cell carcinoma, respectively (*P* = 0.023 and *P* < 0.001, respectively)

The 5-year disease-specific survival of the patients with esophageal small-cell carcinoma was 7% with a median survival time of 15 months. The 5-year disease-specific survival of the patients with high-grade squamous cell carcinoma of the esophagus was 57% (median survival time, 92 months). The survival rate of the esophageal small-cell carcinoma patients was significantly worse than that of the high-grade squamous cell carcinoma patients (*P* < 0.001, Fig. 4). Clinical outcome of the patients with basaloid squamous cell carcinoma (5-year disease-specific survival, 48%) tended to be worse than those with poorly differentiated squamous cell carcinoma (5-year disease-specific survival, 66%), but the difference did not reach statistical significance (*P* = 0.132). Clinical outcome of the small-cell carcinoma (all p16-positive) patients was also significantly shorter than that of p16-positive (*P* = 0.023) as well as that of p16-negative high-grade squamous cell carcinoma patients (*P* < 0.001) (Fig. 4). No significant difference was detected in clinical outcome between the patients with p16-positive and negative high-grade squamous cell carcinoma (*P* = 0.687, Fig. 4).

## Discussion

Esophageal small-cell carcinomas demonstrated consistent overexpression of p16 protein. However, p16 expression was not an exclusive event observed in small-cell carcinoma, also observed in 9% (7/82 cases) of high-grade squamous cell carcinomas. The p16 overexpression was associated with loss of Rb1 protein but not with HPV infection in both esophageal small-cell and high-grade squamous cell carcinomas.

*p16* gene, also known as *CDKN2A*, is frequently inactivated in human malignant neoplasms [9, 10]. The results of a whole-exome sequencing analysis revealed that *CDKN2A* was inactivated in 49–76% of esophageal squamous cell carcinoma and is considered as one of the key events in cancer development [38–40]. *CDKN2A* was inactivated mostly by deletion (44–72%), occasionally by mutation (2–8%). *CDKN2A* DNA hypermethylation could also contribute to its inactivation, and the aberrant methylation of 5′ CpG islands of the *CDKN2A* promoter region leads to repression of its gene transcription [38]. In our present study, we focused on poorly differentiated and basaloid features of squamous cell carcinomas and the results revealed the frequency of p16 protein loss (91%). Of particular interest, Salam et al. reported an association of p16 overexpression with morphological differentiation of the tumor, i.e., loss of p16 expression was more frequently detected in poorly differentiated than in well- and moderately differentiated squamous cell carcinomas [44]. In addition, the status of *p16* promoter methylation was also reported to be associated with histological differentiation, detected in up to 82% of poorly differentiated squamous cell carcinomas [44]. Although the frequency of inactivation of *p16* in basaloid squamous cell carcinomas has not been fully studied at this juncture, an inactivation of the *p16* gene might correlate with high-grade neoplasms. Whether p16 immunoreactivity can serve as a predictive marker for genetic or epigenetic inactivation of *CDKN2A* gene is controversial [41, 42], and further investigations are required for clarification at this juncture [41–43].

Strong nuclear and cytoplasmic expression of p16 observed in continuous areas of the epithelium, called “block” positivity, is typically observed in HPV-associated neoplasms. We have observed this staining pattern in all the small-cell carcinomas included in this study. The p16 overexpression is considered as positive feedback by deregulation of *RB1* [9, 12]. An inactivation of *RB1* was reported to play a pivotal role in carcinogenesis of neuroendocrine carcinomas, and Rb1 protein loss was proposed to be a characteristic in gastroenteropancreatic and pulmonary neuroendocrine carcinomas [6, 14]. In small-cell lung cancer, the mechanism has been shown that at least one allele of *RB1* was affected by different genomic alterations (i.e., hemizygous deletion, loss of heterozygosity, or mutation, including rearrangements) [45]. As small-cell carcinomas of the esophagus and lung greatly share similar biological characteristics, it can be anticipated that these genetic alterations might contribute to the pathogenesis of esophageal small-cell carcinomas [6]. Fujimasa et al. reported that p16 overexpression and Rb1 protein loss were highly specific findings in esophageal small-cell carcinomas and their immunoreactivity could serve as a potentially useful diagnostic marker [12]. In contrast, the p16 expression pattern observed in high-grade squamous cell carcinomas was mostly focal or with a single-cell positivity. Interestingly, 5 out of 6 cases of high-grade squamous cell carcinomas with focal or a single-cell p16 expression also showed loss of Rb1 expression, which might suggest an incomplete activation of p16 protein despite the same p16/Rb1 signaling activation status as observed in small-cell carcinomas.

p16 overexpression in conventional squamous cell carcinoma of the esophagus was reported to be associated with better clinical outcome of the patients [28, 29]. However, in this study, p16 positivity in high-grade squamous cell carcinoma was not associated with tumor size, TNM stage, and patients’ clinical outcome. In the p16-positive high-grade squamous cell carcinoma group, only 4 cases presented the same expression pattern (p16 overexpression/Rb1 protein loss) as that of small-cell carcinomas. Interestingly, the p16 overexpression induced by dysregulation of *RB1* appeared to have an association with tumor aggressiveness in high-grade squamous cell carcinoma. However, due to the limited number of cases available in our present study, further investigations are required for clarification. In addition, considering the high frequency of p16 loss in poorly differentiated squamous cell carcinomas, p16 status is by no means a favorable predictive factor of the patients. The patients with small-cell carcinoma, all of whom expressed p16, had significantly worse survival than those with p16-positive high-grade squamous cell carcinoma despite their common p16/Rb1 signaling activation status. This suggests that the p16/Rb1 signaling might not be a single regulatory mechanism that is associated with the aggressive behavior of the small-cell carcinomas. Due to the limited number of cases, further studies are needed to clarify the prognostic impact of p16 expression in highly malignant esophageal carcinomas. Nevertheless, the results of our present study confirmed the fact that poorly differentiated/basaloid squamous cell carcinomas must be differentiated from esophageal small-cell carcinomas by means of neuroendocrine markers (synaptophysin/chromogranin A) and a squamous-basal marker (e.g., p40).

Diffuse and marked p16 expression usually indicates the presence of HPV infection in squamous cell carcinoma of the uterine cervix, pharynx, and esophagus [15, 16, 28]. Cao et al. reported that the presence of HPV was significantly correlated with p16 immunoreactivity in esophageal squamous cell carcinoma and suggested the p16 immunoreactivity as a good marker for HPV infection [28]. However, p16 can be induced by dysregulation of the RB1 pathway without HPV infection. Indeed, in our present study, HPV infection was detected in none of the p16-positive small-cell and high-grade squamous cell carcinomas. In addition, 5–29% of esophageal squamous cell carcinomas were reported to present p16 expression without association with HPV infection [26, 28, 46, 47]. Based on the results above, immunohistochemical p16 positivity is not indicative of the presence of HPV infection in these highly malignant neoplasms. The reported incidence of HPV infection varies among geographic regions, detection methods, or primary sites. Therefore, further investigations are required for the detection of HPV infection in esophageal neoplasms in larger-scale studies [25–28, 46, 48].

It is also important to note the following limitations in this study. First, PCR to identify HPV genotypes was performed only in limited samples. Second, preoperative treatment might have affected the detection of HPV. Preoperative therapy was performed in some cases because it is the current standard treatment strategy for patients with localized and advanced squamous cell carcinoma of the esophagus in Japan [49]. Third, detection methods of HPV infection in the esophageal tissue have not been fully established. PCR is so far considered to be more reliable than in situ hybridization or immunohistochemistry [48]. These limitations are very hard to avoid in studying with relatively small scale of cases. Nevertheless, we believe despite all above-mentioned limitations, much can be learned from our data.

In summary, p16 overexpression is a constant, but not an exclusive finding in esophageal small-cell carcinomas and could be detected in poorly differentiated and basaloid squamous cell carcinomas albeit with less frequency. p16 overexpression in these highly malignant esophageal neoplasms is considered to represent the sequel of intracellular dysregulation of the RB1 signaling pathway and not HPV infection. The clinical outcome of esophageal small-cell carcinoma patients is significantly worse than that of p16-positive high-grade squamous cell carcinoma patients despite the common p16/Rb1 signaling activation status; however, this has to be further confirmed in a study with a higher number of the relevant neoplasms. Small-cell carcinomas and high-grade squamous cell carcinomas must be, due to the different clinical features, differentiated by neuroendocrine markers (synaptophysin/chromogranin A) and a squamous-basal marker (e.g., p40) expression. In addition, p16 overexpression does not indicate favorable clinical outcome in squamous cell carcinomas with poorly differentiated and basaloid morphology of the esophagus.

## Electronic supplementary material

Supplementary Table 1 Summary of evaluation methods for immunohistochemistry used in this study (XLSX 12 kb)

Supplementary Table 2 Clinicopathological characteristics of 97 patients with esophageal small cell and high-grade squamous cell carcinomas (XLSX 12 kb)

Supplementary Table 3 Comparison of clinicopathological features of p16 positive and negative high-grade squamous cell carcinomas (XLSX 12 kb)

Supplementary Figure 1 Representative illustrations of immunohistochemistry for p16 (a) and Rb1 (b) in basaloid squamous cell carcinoma. p16 expression in non-neoplastic squamous epithelium and several stromal cells (arrowheads, a) served as a positive internal control of p16, whereas carcinoma cells showed negativity for p16 (arrows, a). Nuclear Rb1 immunoreactivity was observed in more than 50% of carcinoma cells with variable intensity (b). Rb1 is also expressed in some stromal cells and non-neoplastic epithelium (b). Histopathological image of cervical intraepithelial neoplasia (c, hematoxylin and eosin staining) and immunohistochemical staining for p16 in the corresponding area (d). Strong nuclear immunoreactivity of p16 was observed in neoplastic cells, whereas majority of non-neoplastic epithelial cells were negative for p16 (d) (PNG 5082 kb)

High resolution image (TIF 1085 kb)
