# Supplementary figures and images for: p16 in highly malignant esophageal carcinomas: the correlation with clinicopathological factors and human papillomavirus infection
Source: Virchows Arch. 2020 Jun 16;478(2):219–29. doi: 10.1007/s00428-020-02865-x (PMC7969492; doi:10.1007/s00428-020-02865-x)

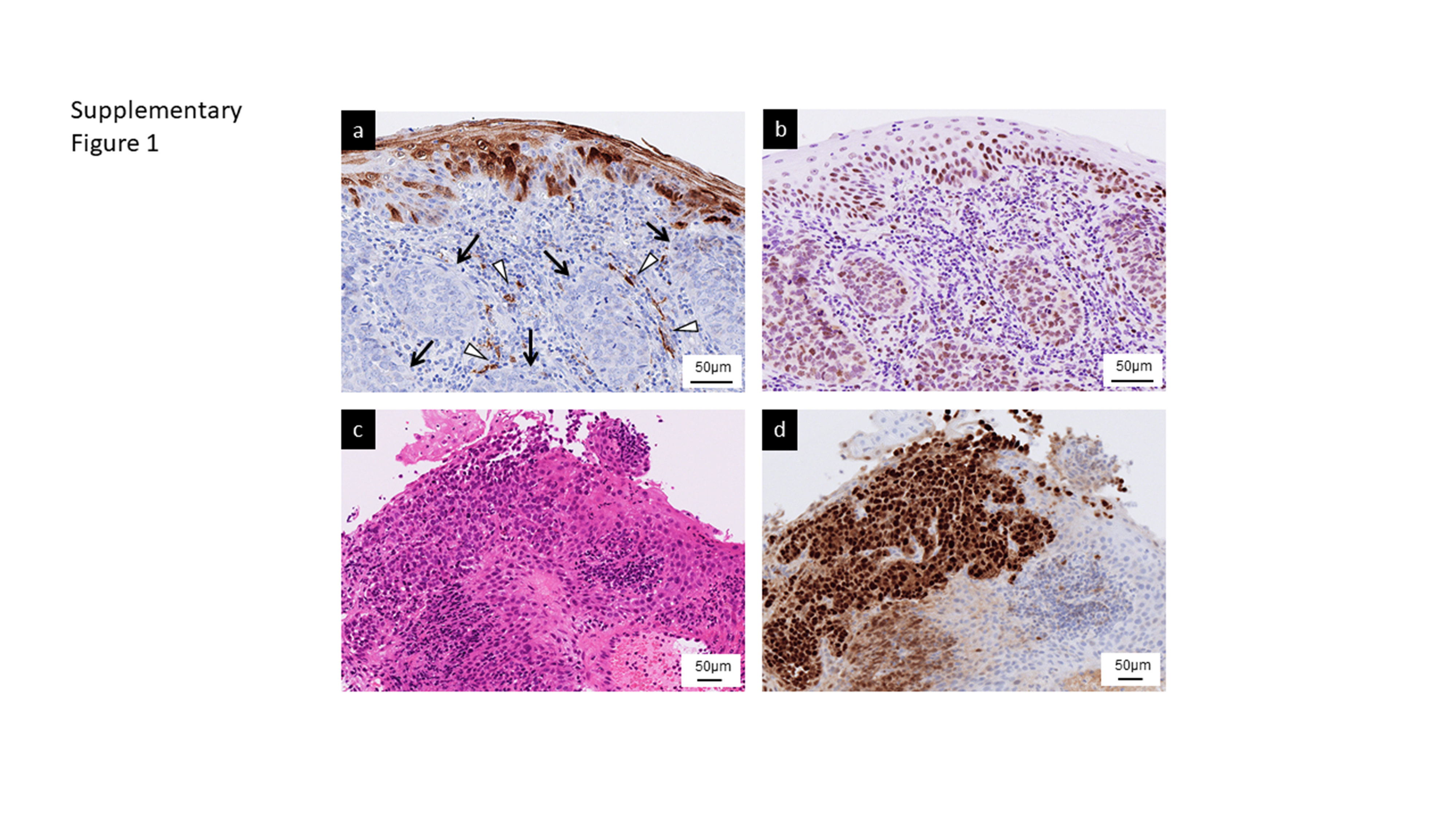

Supplement: Supplementary file 4 — Representative illustrations of immunohistochemistry for p16 (a) and Rb1 (b) in basaloid squamous cell carcinoma. p16 expression in non-neoplastic squamous epithelium and several stromal cells (arrowheads, a) served as a positive internal control of p16, whereas carcinoma cells showed negativity for p16 (arrows, a). Nuclear Rb1 immunoreactivity was observed in more than 50% of carcinoma cells with variable intensity (b). Rb1 is also expressed in some stromal cells and non-neoplastic epithelium (b). Histopathological image of cervical intraepithelial neoplasia (c, hematoxylin and eosin staining) and immunohistochemical staining for p16 in the corresponding area (d). Strong nuclear immunoreactivity of p16 was observed in neoplastic cells, whereas majority of non-neoplastic epithelial cells were negative for p16 (d) (PNG 5082 kb) [file 428_2020_2865_Fig5_ESM.png]

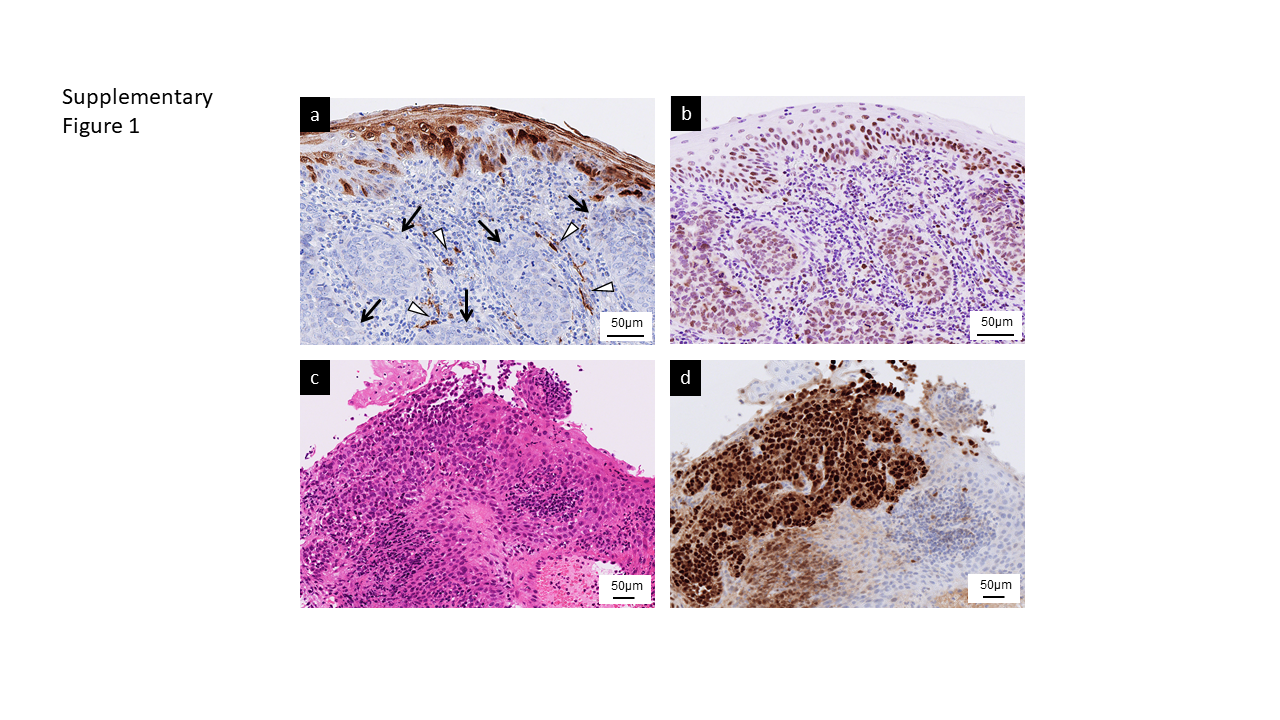

Supplement: Supplementary file 5 — High resolution image (TIF 1085 kb) [file 428_2020_2865_MOESM4_ESM.tif]
